# Effects of a Macromolecule Spirocyclic Inflatable Flame Retardant on the Thermal and Flame Retardant Properties of Epoxy Resin

**DOI:** 10.3390/polym12010132

**Published:** 2020-01-06

**Authors:** Kunpeng Song, Yinjie Wang, Fang Ruan, Jiping Liu, Nianhua Li, Xueli Li

**Affiliations:** School of Materials Science and Engineering, Beijing Institute of Technology, 5 Zhongguancun South Street, Haidian District, Beijing 100081, China; 13439970074@163.com (K.S.); 18255171997@163.com (F.R.); 17888819050@163.com (N.L.); 15733185216@163.com (X.L.)

**Keywords:** spirocyclic, intumescent flame retardant, epoxy resin, flame retardant mechanism

## Abstract

A new strategy for the preparation of an integrated three-source intumescent flame retardant (IFR) has been developed to improve the flame-retardant and smoke suppression performance of epoxy resin (EP) with a synergistic flame retardant effect. Herein, the synthesis of a macromolecular spirocyclic phosphorus/nitrogen-containing IFR poly sulfonamide spirocyclic pentaerythritol bisphosphonate (SAPC) is reported via a two-step method that uses pentaerythritol, phosphorus oxychloride and sulfonamide (SAA) as raw materials. Subsequently, the SAPC was incorporated into EP to prepare the composite to investigate its thermal stability, flame retardancy, and smoke suppression performance. Herein, a differential scanning calorimetry (DSC) analysis showed that the addition of SAPC increased the glass transition temperature (*T*_g_) of the composite. Cone test results indicated that the incorporation of 8 wt % SAPC significantly improved the flame-retardant performance for the composite, with a 43.45% decrease in peak of heat release rate, a 28.55% reduction in total heat release, and a 30.04% decrease in total smoke release. Additionally, the composite received the V-0 rating in a UL-94 vertical burning test, accompanied by the “blowout” phenomenon. After the addition of SAPC, the amount of flammable gas products from the EP composite decomposition was obviously suppressed, and the amount of non-flammable as was increased. All of this suggests a good dilution role of SAPC. There are enough reasons to believe that the enhanced flame-retardant and toxicity suppression performance for the EP composite can be attributed to the good coordination of carbonization agent, acid source, and blowing agent in the SAPC structure.

## 1. Introduction

The industrial production of epoxy resin (EP) has been in practice for more than 60 years. EP is a kind of multi-purpose resin that is widely used in various industrial fields such as adhesives, transportation, aerospace, electronics, and composite materials [1,2,3]. However, the limiting oxygen index (LOI) of unmodified EP is only 19.8%, accompanied by more severe smoke during the burning process [4,5,6]. This defect seriously hinders EP’s development and application [7]. The incorporation of flame retardants into EP can effectively alleviate this situation [8,9,10].

With the revision and promulgation of the “Waste Electrical and Electronic Equipment” and the “Restriction of Hazardous Substances” directives, the use of traditional halogen-containing flame retardants have been greatly restricted. However, the intumescent flame retardant (IFR) is considered to be an important way to make the flame retardant process halogen-free and green [11]. Since the 1990s, the flame retardant mechanism of IFRs has been continuously updated, and modified IFR systems have also emerged [12,13].

Phosphate flame retardants have the advantages of low smoke, low toxicity, relatively high flame retardant efficiency, and good compatibility with a matrix, thus presenting a good development prospects [14,15,16,17,18]. Compared to aliphatic phosphonates, spirocyclic phosphates show a superior flame retardant efficiency due to their rich carbonization agent and phosphorus content. SPDPC (3,9-dichloro-2,4,8,10-tetraoxa-3,9-diphosphaspiro-[5,5] undecane-3,9-dioxide) is prepared through the nucleophilic substitution of pentaerythritol and phosphorus oxychloride, and it is a flame retardant intermediate that is widely used in spirocyclic phosphates. It is a double-ring rigid structure with high symmetry, which gives it a high thermal stability [19,20]. SPDPC is a bifunctional compound with highly reactive P–Cl bonds at both ends, and these can be used as additive flame retardant by themselves [21], as well as intermediates to react with amino compounds containing amino (–NH_2_) or imino groups (–NH), thus obtaining IFRs that are integrated with an acid source, a carbonization agent, and a blowing agent [22,23,24,25].

In this past decade, the preparation of the spirocyclic IFRs that integrate three sources has attracted increasing interest. Jiang reported the preparation of the flame retardant imidazole spirocyclic phosphoramidate (ISPA) by the substitution reaction of SPDPC and imidazole for flame retardant cotton fabrics. However, there are problems in this process, such as a high amount of addition and an increase in the amount of smoke released during combustion [26]. In this regard, it is necessary to further study the flame retardant and smoke suppression functions of IFRs. Many studies have shown that macromolecular IFRs tend to exhibit a better comprehensive performance than small IFRs [27,28,29]. Wu prepared a phosphorus–nitrogen-containing IFR poly ethanediamine spirocyclic pentaerythritol bisphosphonate (PEPS) by the reaction of SPDPC and ethylenediamine to create a flame retardant rigid polyurethane foam. The results showed that the composite presented good flame retardancy and water resistance. In addition, PEPS was uniformly dispersed in the matrix, and its mechanical property was less lost than usual [28]. Su synthesized a novel reactive polyphosphamide flame retardant poly 4,4-diaminodiphenylsulfone spirocyclic pentaerythritol bisphosphonate (PCS) through the polycondensation of SPDPC with 4, 4-diaminodiphenyl sulfone. The prepared EP composite exhibited a good flame retardant effect, but the addition amount was relatively high [21]. Zhao synthesized a novel organophosphorus polymeric flame retardant poly (hydroxyphenyl imino methyl phenol spirocyclic pentaerythritol diphosphonate) (PPISP) for unsaturated polyester resin (UP) that contained both a Schiff base and spirocyclic diphosphate structures. The prepared UP/PPISP showed excellent flame-retardant durability and water resistance. Moreover, this study also enriched the research on functional flame retardants [30]. Recently, a kind of intumescent, flame-retardant curing agent poly-(cyclohexane-1, 3-diyldimethanamine spirocyclic pentaerythritol bisphosphonate) (PCDSPB) that endowed a distinguished flame-retardant property in the EP composites, even at a low phosphorus content, was synthesized. However, compared with pure epoxy resin, the initial decomposition temperature (*T*_5%_) of PCDSPB was relatively low at 282 °C, which resulted in a significant reduction in the thermal stability of the epoxy composite [31]. Layered double hydroxide (LDH) exhibits a high anion-exchange capacity and a good thermal stability, and these properties have also provided new ideas for the flame retardant field. Previous studies have shown that proper modification of LDH can help improve the flame retardancy of composites [32,33]. Studies on employing SPDPC to improve LDH dispersion and flame retardancy have been reported via the hydrothermal method based on SPDPC and Mg–Al–LDH (N–LDH). Moreover, this flame retardant modification can also effectively improve the mechanical properties of nanocomposites [34].

On the whole, IFRs generally have problems such as low flame retardant efficiency and a relatively ineffective smoke suppression effect. At present, many IFRs reduce the glass transition temperature of EP composites while exerting flame retardant effects, resulting in restrictions in electronic packaging and other fields. In addition, there are many reports on IFRs, but investigations of their flame retardant mechanism are generally not profound. To this end, this paper introduces a spirocyclic IFR SAPC to prepare EP composites. The thermal stability, flame retardancy, and flame retardant mechanism of EP composites were analyzed.

## 2. Experimental Section

### 2.1. Materials

The pentaerythritol (≥98%), phosphorus oxychloride (≥98%) and glacial acetic acid (98%) used in this study were obtained from the Tianjin Nankai Yungong Synthesis Technology Co., Ltd. (Tianjin, China). Acetonitrile (AR) and 4, 4-diaminodiphenylmethane (≥98%) were purchased from the Beijing Chemical Plant (Beijing, China). Acetone, dichloromethane, and triethylamine were provided by Weiss Chemical Reagent Co., Ltd. (AR, Beijing, China). Sulfonamide (98%) was purchased from the Tianjin Komiou Chemical Reagent Co., Ltd. (Tianjin, China). The diglycidyl ether of bisphenol A (E-44, epoxy equivalent = 0.44 mol/100 g) was procured from Nantong Xingchen Synthetic Material Co., Ltd. (Hunan, China). Acetonitrile and triethylamine (TEA) were dried over 4 Å molecular sieves before use.

### 2.2. Synthesis of SAPC

**Scheme 1:**
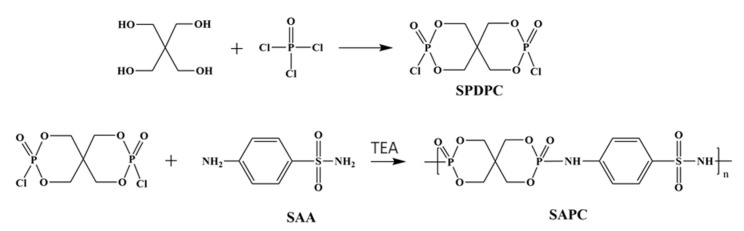
Synthetic route of SPDPC (3,9-dichloro-2,4,8,10-tetraoxa-3,9-diphosphaspiro-[5,5] undecane-3,9-dioxide) and SAPC.

The preparation of the flame retardant SAPC was divided into two steps. First, the intermediate SPDPC was synthesized, and then it was reacted with sulfonamide (SAA) to obtain SAPC. The synthetic route is illustrated in Scheme 1.

In the first step, pentaerythritol (20.4 g; 0.15 mol) was added into 100 mL of anhydrous acetonitrile and then stirred for 20 min at 55 °C under a nitrogen atmosphere. Then, phosphorus oxychloride (69.3 g; 0.45 mol) was added to the above solution and stirred for 2 h at 60 °C. After the reaction temperature went up to 80 °C, the reaction was continued until no HCl gas was generated. Subsequently, the mixture was filtered, and the filter cake was collected was washed 3 times with acetone and dichloromethane to obtain a white powder. Finally, the powder was recrystallized in glacial acetic acid to obtain a higher purity intermediate SPDPC in a yield of 71.6%.

In the second step, sulfonamide (5.424 g; 0.0315 mol) was dissolved in 100 mL of anhydrous acetonitrile under a nitrogen atmosphere. Then, SPDPC (8.94 g; 0.03 mol) was added to the above solution. After stirring for 20 min, 8.4 mL of triethylamine (TEA) was added. The mixture was stirred for 2 h at 60 °C and then stirred for another 12 h with the reaction temperature up to 80 °C. Then, the product was obtained with a filter and was washed three times with acetone and dichloromethane, successively. Finally, it was dried at 80 °C in a vacuum oven to obtain SAPC in a yield of 78.4%.

### 2.3. Preparation of the Composites EP/SAPC

**Scheme 2:**
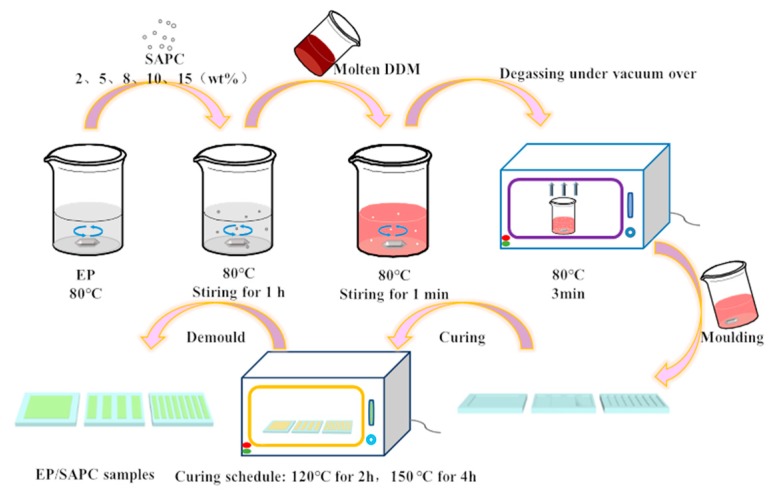
Schematic of the preparation of the epoxy resin (EP)/SAPC composites.

Different mass fractions of SAPC (0, 2, 5, 8, 10, and 15 wt %) were first dispersed in epoxy resin while stirring at 80 °C for 1 h. The hardener 4,4-diaminodiphenylmethane (DDM) (ratio of DDM/epoxy was 1:4) was then added to the above solution. The mixture was stirred at 80 °C for 1 min to form a homogeneous liquid, which was transferred into the vacuum oven at 80 °C for 3 min to remove bubbles; it was then immediately poured into pre-heated molds of certain sizes. In the final step, the epoxy mixtures were cured in the blast oven at 120 °C for 2 h, and then they were heated to 150 °C for 4 h to obtain EP composites. The prepared samples were denoted as EP control, EP/SAPC-2, EP/SAPC-5, EP/SAPC-8, EP/SAPC-10, and EP/SAPC-15. The schematic diagram of the preparation method is shown in Scheme 2.

### 2.4. Characterization

Fourier-transform infrared (FTIR) spectroscopy: FTIR spectra were recorded on a Tensor 27 IR spectrometer (BRUKER OPTICS, Beijing, China). Spectra were collected at 32 scans with a spectral resolution of 4 cm^−1^, and the test range was 500~4000 cm^−1^.

Nuclear magnetic resonance (NMR) spectroscopy: ^1^H-NMR, ^13^C-NMR and ^31^P-NMR spectra were recorded on an FT-80A NMR spectrometer (VARIAN, Palo Alto, CA, USA). (Methyl sulfoxide)-d6 (DMSO-d6) was used as the solvent, and the solution was measured with tetramethyl silane (TMS) and phosphoric acid as internal standards.

Gel permeation chromatography (GPC): Waters Breeze 2 high performance liquid chromatography (Waters, Milford, MA, USA) was employed to detect the flame retardant SAPC. The mobile phase was tetrahydrofuran, and the solvent elution rate was 0.5 mL/min. During the detection, the column and detector were kept at a constant temperature of 40 °C.

Thermogravimetric analysis (TGA) and differential scanning calorimetry (DSC) were performed with a TOLEDO STARE thermal analyzer (Mettler-Toledo, Zurich, Switzerland). Measurements were carried out in a nitrogen and air atmosphere from 50 to 800 °C at a heating rate of 20 °C/min and from 50 to 180 °C with a heating rate of 5 °C/min under a nitrogen atmosphere, respectively.

Limiting oxygen index (LOI) tests were performed with an FTAII 1600 LOI instrument (Phoenix Instruments Co., Ltd., Suzhou, China) by using the standard ASTM D 2863 procedure. The sample dimensions were 130 × 6.5 × 3 mm^3^.

Vertical burning tests were performed with a CZF-5 horizontal vertical burning tester (Phoenix Instruments Co., Ltd., Suzhou, China) with the UL-94 standard on samples with dimensions of 130 × 13 × 3 mm^3^.

Cone calorimeter measurements were performed with a Fire Testing Technology (FTT) apparatus (Phoenix Instruments Co., Ltd., Suzhou, China) with a truncated cone-shaped radiator, and the measurements taken according to the ISO 5660 protocol with an incident radiant flux of 50 kW/m^2^. The specimen (100 × 100 × 3 mm^3^) was measured horizontally without any grids. The reported results were averaged from two measurements.

Scanning electron microscopy (SEM) was performed with a Hitachi SU8020 (Hitachi Limited, Tokyo, Japan) with a 15 kV accelerating voltage. The samples were sprayed with a thin gold layer to make good electrical surface conductivity.

The X-ray photoelectron spectroscopy (XPS) patterns of the samples were obtained by using Quantera II X-ray photoelectron spectroscopy (Ulvac-PHI, Chigasaki, Japan), and the measurements were carried out at 25 W power and 10^−6^ Pa vacuum.

The thermogravimetric analysis (Mettler-Toledo, Zurich, Switzerland) was coupled with Fourier-transform infrared spectroscopy (BRUKER OPTICS, Beijing, China), and this was carried out under a nitrogen atmosphere from 50 to 800 °C at a heating rate of 20 °C/min^−1^.

## 3. Results

### 3.1. Characterization of the Structure and Thermal Stability of SAPC

**Figure 1:**
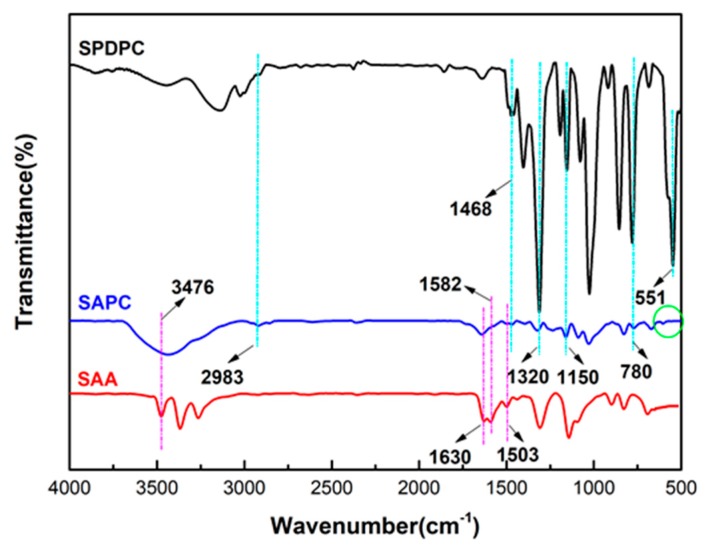
Fourier-transform infrared (FTIR) spectroscopy.

FTIR spectra were measured to confirm the emergence of a polycondensation reaction between the SPDPC and SAA. As shown in Figure 1, the typical peaks of the SAPC and SPDPC spectra at 2983 and 1468 cm^−1^ corresponded to –CH_2_– stretching and bending vibrations; three apparent peaks at 1320, 1150, and 780 cm^−1^ were attributed to P=O, P–O–C and pentaerythritol carbon skeleton stretching vibration, respectively. The existence of these absorption peaks proves that SAPC retained the basic structure of SPDPC. The sharp peaks at 551 cm^−1^ corresponding to the stretching vibration of the P–Cl bond and the multiple absorption peaks at 3250–3500 cm^−1^ were assigned to the amino in SAA units. For the SAPC spectrum, there was no significant P–Cl absorption peak at 551 cm^−1^, but a new secondary amino group appeared at 3476 cm^−1^. Additionally, three apparent peaks at 1630, 1582, and 1503 cm^−1^ corresponded to the benzene ring structure in SAA units. Based on these results, the P–Cl in the SPDPC units completely reacted with the –NH_2_ of the SAA units [35,36].

**Figure 2:**
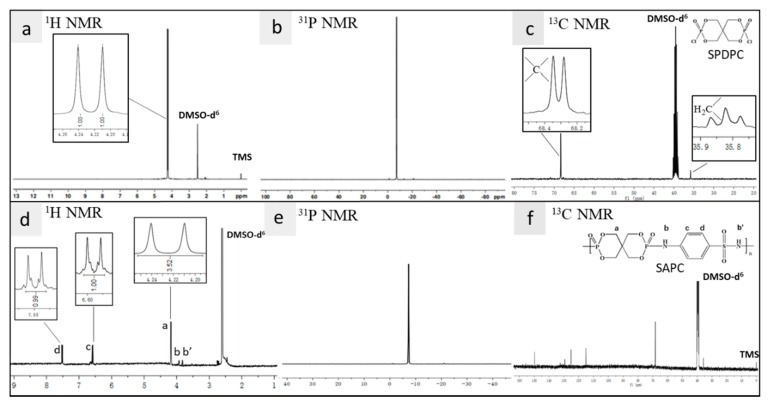
^1^H NMR (**a**), ^31^P NMR (**b**) and ^13^C NMR (**c**) spectra of SPDPC; ^1^H NMR (**d**), ^31^P NMR (**e**) and ^13^C NMR (**f**) spectra of SAPC.

The ^1^H NMR, ^31^P NMR and ^13^C NMR spectra of SPDPC and SAPC are presented in Figure 2. The characteristic peaks with two equal heights at 4.21 and 4.24 ppm were resonance peaks of the methylene groups (Figure 2a), and the ratio of protons was approximately 1:1, which proves that a spirocyclic existed in the structure. As can be seen in Figure 2b, there was only one single peak at −7.2 ppm, and this corresponded to the resonance peak of phosphorus of SPDPC. In addition, the peaks at δ = 35.7–35.9 ppm could be assigned to quaternary carbon atom in the spirocyclic moiety, and the peaks at 68.20–68.4 corresponded to methylene of the spirocyclic moiety (Figure 2c). In summary, this all shows that SPDPC was successfully obtained.

As shown in Figure 2d, the resonance peaks at 4.20 and 4.25 ppm corresponded to methylene. Two characteristic protons of the aromatic appeared at 6.55–6.65 and 7.50–7.60 ppm. In addition, the resonance peaks at 3.8 and 3.9 ppm were assigned to N–H. The ^31^P NMR spectra of both SAPC and SPDPC had only one resonance peak, indicating that phosphorus was in the same chemical environment (Figure 2e). As can be seen in Figure 2f, SAPC had benzene-carbon resonance peaks at 115–150 ppm. Based on these results, SAPC was effectively synthesized.

**Figure 3:**
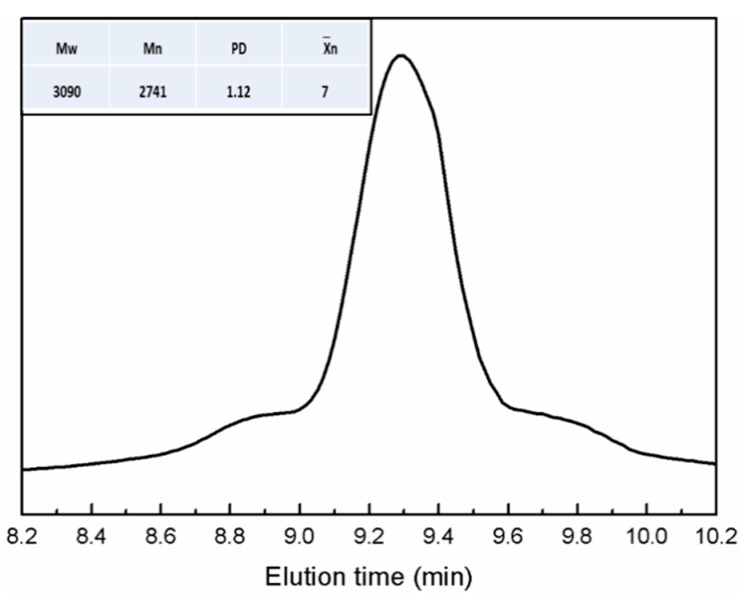
Gel permeation chromatography (GPC) curve of SAPC.

Moreover, the molecular weight and molecular weight distribution of the flame retardant SAPC were characterized by GPC (Figure 3). SAPC presented a relatively uniform molecular weight distribution, and the degree of polymerization (*X*_n_) was calculated to be approximately 7.

### 3.2. Analysis on Thermal Properties of EP/SAPC Composites

**Figure 4:**
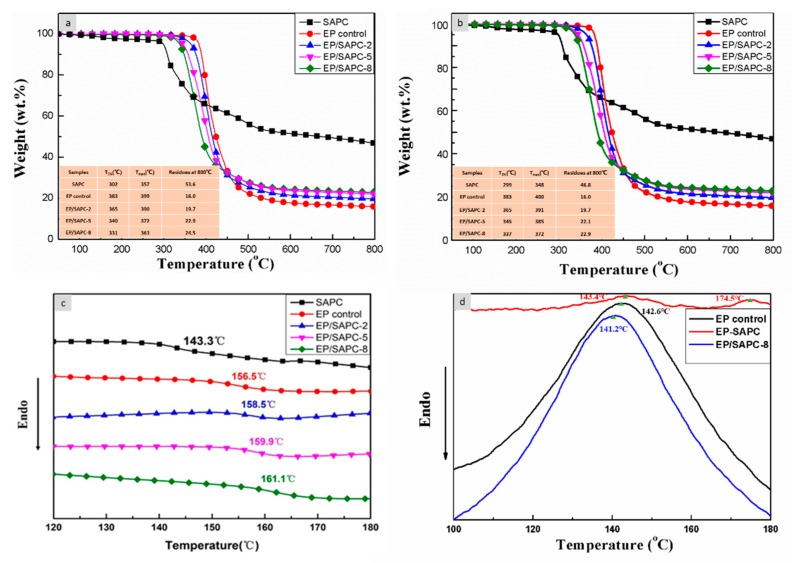
(**a**) Thermogravimetric analysis (TGA) curves under the nitrogen atmosphere; (**b**) TGA curves under the air atmosphere; (**c**) differential scanning calorimetry (DSC) curves; (**d**) DSC curves during curing process.

As shown in Figure 4a, SAPC presented a one-step thermal decomposition process with approximately 53.6% residual char at 800 °C under the nitrogen atmosphere, mainly ascribed to the degradation of the macromolecular chains, indicating that SAPC showed a good thermal stability and char forming ability. For both the EP/SAPC composites, the onset decomposition temperature (*T*_5%_, the temperature at 5% mass loss) and the maximum decomposition temperature (*T*_max_, the temperature at maximum mass loss) were lower than that of the EP control due to the faster thermal degradation of SAPC and the catalytic effect of phosphorus and nitrogen. As far as the residual char yield is concerned, SAPC resulted in the improvement of residues at 800 °C, which could possibly be ascribed to SAPC’s function as a barrier that inhibited the complete decomposition of the epoxy molecular chain. The acid source of SAPC decomposed to form a polyphosphoric acid compound, which promoted the dehydration carbonization of epoxy molecular chain and reduced the loss of decomposition products from the condensed phase to the gas phase [37,38,39]. Moreover, the thermal decomposition behavior of the SAPC and EP/SAPC composites under the air atmosphere was the same as that under the nitrogen atmosphere (Figure 4b).

**Scheme 3:**
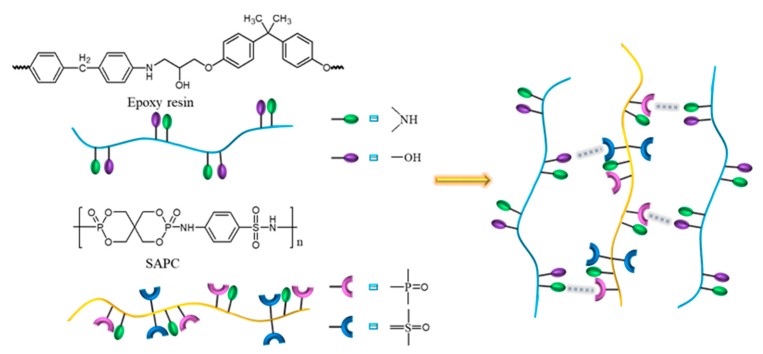
Schematic of possible hydrogen bonding between SAPC and EP.

The EP/SAPC composites showed only one *T*_g_, thus indicating that SAPC had a good compatibility with EP (Figure 4c). For both the EP/SAPC composites, the *T*_g_ was slightly higher than the EP control, and the improvement was more obvious with the increase of SAPC content, which could mainly be attributed to two factors. On the one hand, the amino groups of the SAPC participated in the curing reaction, increasing the crosslinking density of the epoxy molecular chain. On the other hand, the hydrogen bonding interaction between SAPC and the epoxy molecular chain further limited the movement of molecular segments [40]. Scheme 3 depicts the schematic of the possible hydrogen bonding between SAPC and EP.

To further study the influence of the SAPC on the curing reaction, experiments regarding the curing process of the EP control, the mixture of SAPC and epoxy resin (EP-SAPC) without the curing agent DDM (the ratio of SAPC/epoxy resin was 2:23), and EP/SAPC-8 DSC were performed. From Figure 4d, it can be seen that the EP control and EP/SAPC-8 showed obvious exothermic peaks around 140 °C, which was due to the curing reaction between DDM and epoxy resin. However, for EP-SAPC, exothermic peaks could be observed at 143.4 and 174.5 °C, the latter of which was more pronounced. Therefore, it could be proven that SAPC can react with the epoxy resin during the curing process.

### 3.3. Analysis on Fire Safety of EP/SAPC Composites

**Figure 5:**
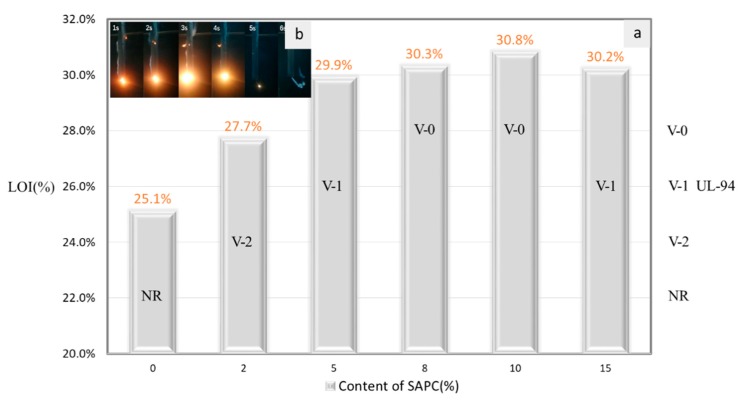
(**a**) The limiting oxygen index (LOI) and vertical combustion analysis of EP/SAPC composites; (**b**) “blowout” digital photos in vertical burning test.

The fire behaviors of the EP composites were evaluated by using the LOI and UL-94 tests [41]. From Figure 5a, it can be seen that the addition of SAPC increased the LOI values of the EP composites. When the added amount of SAPC was increased from 0 to 8 wt %, the LOI of the composite increased from 25.1% to 30.3%, reaching the V-0 rating with the “blowout” phenomenon (Figure 5b). Interestingly, when the amounts of SAPC were increased from 10 to 15 wt %, the LOI values and the UL-94 rating of the composites decreased to some extent, which may have been due to a strong gas flow that destroyed the integrity of the char layer. A cone calorimeter test was performed on these samples to further investigate their fire performance.

**Figure 6:**
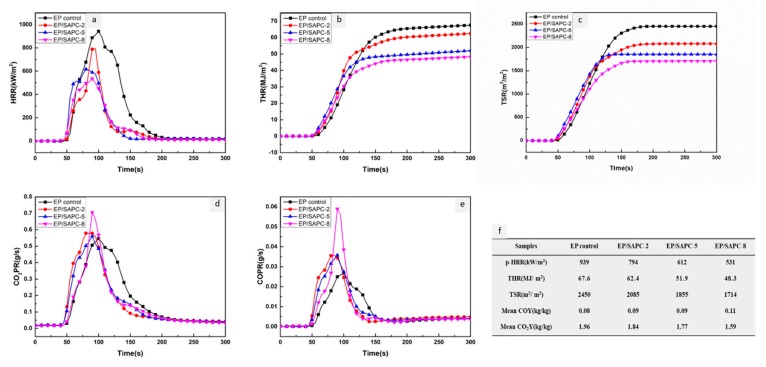
(**a**) Heat release rate (HRR); (**b**) total heat release (THR); (**c**) total smoke release (TSR); (**d**) CO_2_ production release rate (CO_2_PR) curves; (**e**) CO production release rate (COPR) curves; (**f**) analysis data of EP/SAPC composites.

Heat release rate (HRR), total heat release (THR), and total smoke release (TSR) curves of the EP control and its composites were obtained from cone calorimeter (Figure 6). Compared to the EP control, EP/SAPC-8 resulted in about a 43.45% maximum decrease in HRR, a 28.55% maximum decrease in THR, and a 30.04% maximum decrease in TSR. This was mainly attributed to the phosphoric acid that was produced by the early decomposition of SAPC and that promoted the dehydration of the epoxy molecular chain to form a dense char layer [42,43,44].

From the results shown in Figure 6f, it can be seen that the amounts of the mean CO_2_ yield (mean CO_2_Y) of EP/SAPC-8 decreased from 1.96 to 1.59, and that of the mean CO yield (mean COY) increased from 0.08 to 0.11. This was closely related to the phosphorus-containing free radicals that were produced by the SAPC decomposition. Phosphorus-containing free radicals captured active radicals such as H•, HO• and O• in the combustion chain reaction, exerting a quenching effect. Incomplete combustion resulted in a decreased mean CO_2_Y and an increased mean COY from combustion [45]. Interestingly, the mean CO_2_Y tended to decrease with the increasing SAPC content. However, the peak value of the CO_2_PR curve was the same as the peak of the COPR curve. Compared with the EP control, the peaks of CO_2_PR and COPR for EP/SAPC-8 increased by 29.63% and 126.92%, respectively. The above results reveal that the “blowout” phenomenon of EP/SAPC-8 in the UL-94 test was closely related to the large amount of CO and CO_2_ generated in a short time.

### 3.4. Analysis on the Flame-Retardant Mechanism of Condensed Phase for EP/SAPC Composites

**Figure 7:**
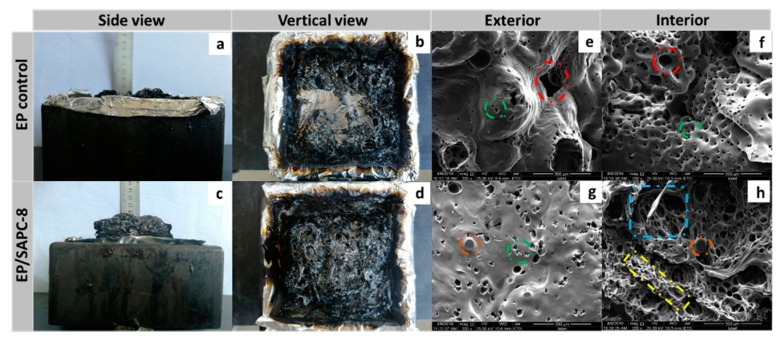
Digital photos (**a**–**d**) and SEM images (**e**–**h**) of the char residues from the EP control and EP/SAPC-8, respectively.

The char residues of the EP control and its composites after cone calorimeter tests were compared from different visual angles (Figure 7a–d). It can be seen that the EP control was completely burned. However, the char residues of EP/SAPC-8 appeared to be more intumescent and compact with a hard surface, showing a better integrity than the EP control.

The microstructures of char layers are shown in Figure 7e–h. The exterior and interior char layers of EP/SAPC-8 were more compact and continuous than that of the EP control, and these layers could have functioned as physical barriers. In particular, quite small pores were formed in the exterior char layers of EP/SAPC-8 after burning, which may have been due to a strong airflow. It could be seen that, in accordance with the HRR, THR and TSR curves, honeycomb-shaped and a certain thickness of “foam” char layers were formed in the interior char layers, indicating that heat and smoke were gently released through these channels [46,47]. The honeycomb char structure acted as a good physical barrier to seal most of the smoke and flue gas that were generated by combustion, reducing the exchange rate of heat, combustibles and oxygen to the substrate [48,49].

**Figure 8:**
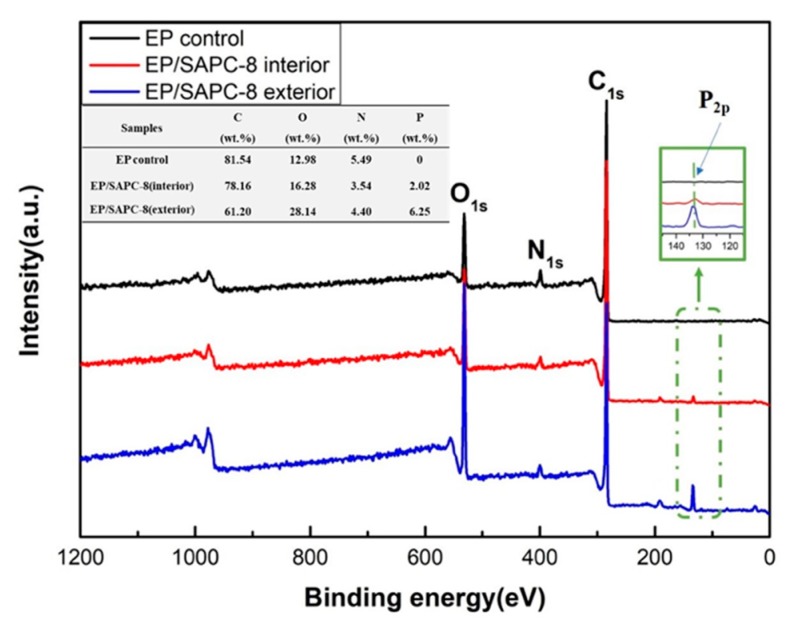
X-ray photoelectron spectroscopy (XPS) spectrum of the EP control and EP/SAPC-8.

The interior and exterior char residues of the EP control and EP/SAPC-8 after cone calorimeter tests were further observed and compared by XPS (Figure 8). The EP/SAPC-8 char residues presented a distinct characteristic peak of the P element at 139 eV, and its exterior char layer was stronger than that of the interior char layer, implying that the phosphorus-containing compound that was produced by the combustion tended to migrate to the exterior. In addition, the O content of EP/SAPC-8 residual char was significantly increased, especially for exterior char layer, when compared to the EP control. On the one hand, this could be ascribed to the fact that the introduced SAPC contained a certain amount of O elements. On the other hand, the P element and the O element formed phosphorus-containing compounds such as phosphoric acid and metaphosphoric acid in the form of OP^+^, O_2_P^+^ and HO_2_P^+^ [50]. This could also be explained by the fact that the P and O contents in the exterior char layer were higher than those in the interior char layer.

### 3.5. Analysis of Gas Phase Flame Retardant Mechanism of EP/SAPC Composites

**Figure 9:**
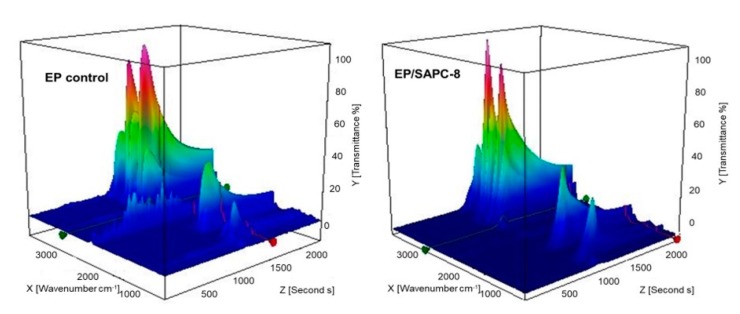
Three-dimensional (3D) TG-FTIR spectra of gasified pyrolysis products for the EP control and EP/SAPC-8.

TG-FTIR was performed to further analyze the gaseous pyrolysis products that were generated during thermal decomposition process. As shown in Figure 9, 3D TG-FTIR spectra were obtained during the thermal decomposition of the EP composites. It is obvious that the peak intensity of EP/SAPC-8 was reduced and reached a steady state in advance, as compared to the EP control.

**Figure 10:**
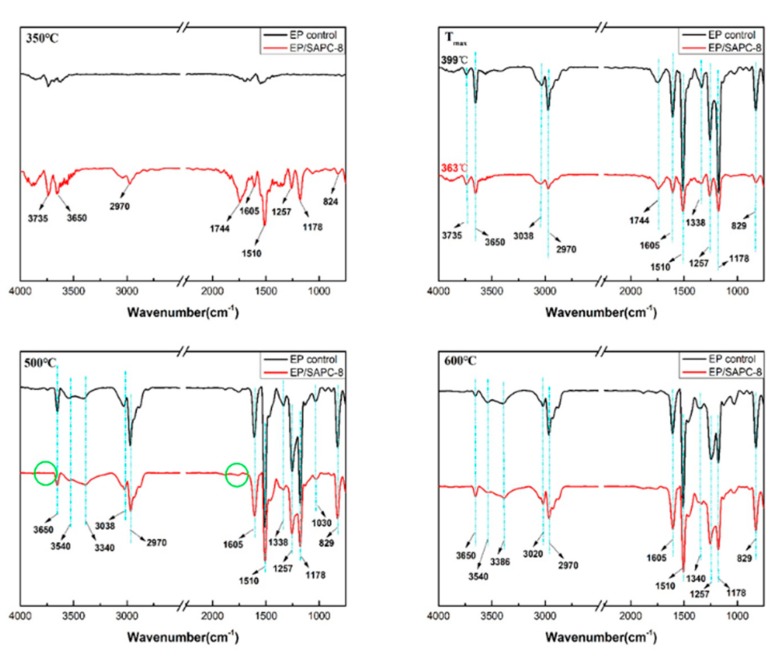
FTIR spectra of the pyrolysis products of the EP control and EP/SAPC-8 at 350 °C, T_max_, 500 °C, and 600 °C.

The FTIR spectra of the pyrolysis products for the EP control and EP/SAPC-8 at different temperatures are shown in Figure 10, and the corresponding data are collected in Table 1. Several flammable and non-flammable gaseous products of EP/SAPC-8 were weaker than the EP control, as identified by characteristic FTIR signals after *T*_max_. However, the EP/SAPC-8 started to release the pyrolysis products slightly earlier than the EP control, indicating that addition of SAPC catalyzed the thermal degradation of EP. It is worth noting that the absorption peaks at 3735 and 1744 cm^−1^ had almost disappeared at 500 °C, indicating that the amount of the water or phenolic compounds was decreased. Meanwhile, the carbonyl compound was also substantially completely released. At 600 °C, there was no significant difference between the infrared absorption peaks of the two, indicating that the decomposition tended to be stable.

**Figure 11:**
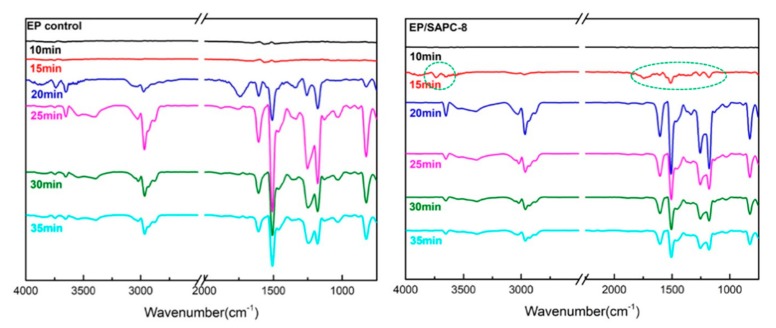
FTIR spectra of the EP control and EP/SAPC-8 at different time periods.

The FTIR spectra of the representative pyrolysis products for EP and EP/SAPC at different times are presented in Figure 11. Comparing the decomposition processes of the EP control and EP/SAPC-8, the positions of the absorption peaks were basically the same, suggesting that the pyrolysis products were basically consistent. Differently, EP/SAPC-8 decomposed in advance and reaches a stable state earlier.

**Figure 12:**
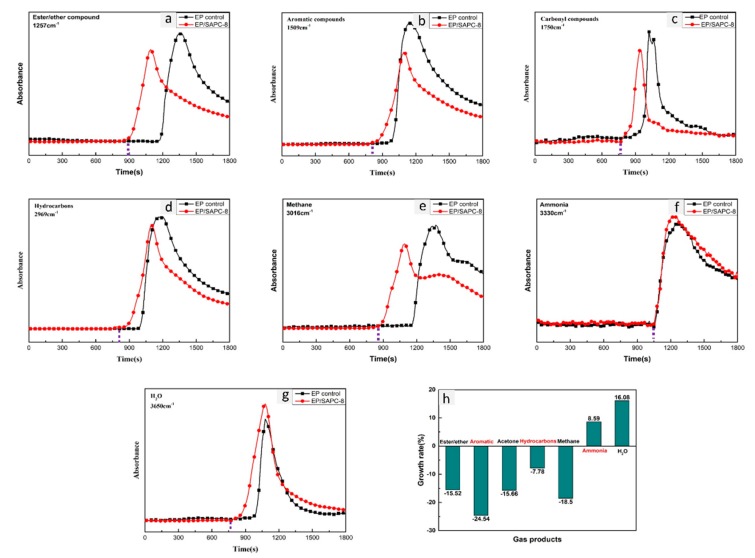
Absorbance of pyrolysis products for EP control and EP/SAPC-8 vs. time: (**a**) esters/ethers, (**b**) aromatic compounds, (**c**) carbonyl compounds, (**d**) hydrocarbons, (**e**) methane, (**f**) ammonia, (**g**) H_2_O, (**h**) various gas pyrolysis product growth rates.

In order to make a further comparison, the FTIR spectra of representative pyrolysis products were obtained for the composites (Figure 12a–g). Regarding the decomposition time, the carbonyl compound was first released due to the lower C=O bond energy in the crosslinked network. Next, the aromatic compound, the fatty chain compound, methane, and water vapor were successively released. Finally, the decomposition products were esters/ethers and ammonia.

With the incorporation of 8% SAPC, the maximum absorbance intensity of the representative pyrolysis products—esters/ethers compounds, aromatic and carbonyl compounds, hydrocarbons, and methane—was shifted to a lower value than that of the EP control sample. In addition, the introduction of SAPC advanced the release time of the pyrolysis volatiles. This was inextricably linked to the early decomposition of SAPC and its catalytic action. Moreover, there were significant difference for H_2_O and ammonia, which may have been due to the fact that SAPC changed the decomposition path of the EP to some extent. It is worth noting that the rising H_2_O and ammonia content diluted the concentration of combustible gas (Figure 12h), which was beneficial for the improvement of the flame retardant effect. The EP/SAPC composites achieved the purpose of suppressing combustion by increasing the release of non-combustible gas and reducing the generation of combustible gas.

**Scheme 4:**
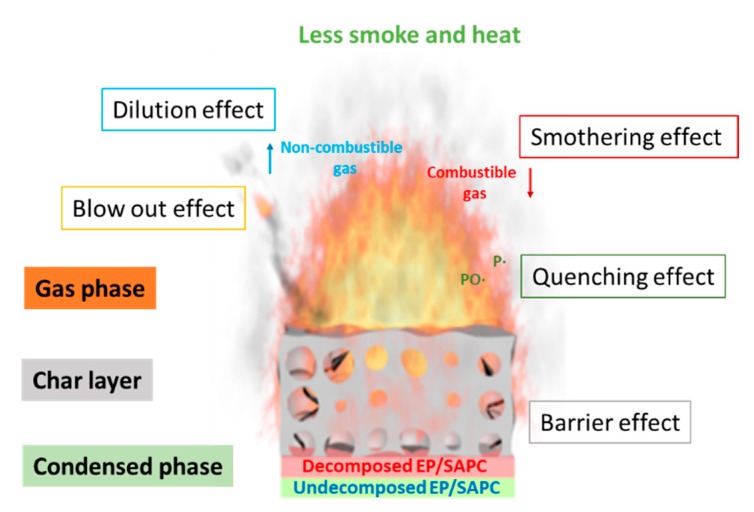
Flame retardant mechanism for the EP/SAPC composites.

A flame retardant mechanism for the EP/SAPC composites was speculated based on the analyses of char residues and pyrolysis products (Scheme 4). In the condensed phase, the phosphorus-containing compounds that were produced by the SAPC decomposition catalyzed the formation of the char residues. In addition, the pyrolysis volatiles participated in the foaming role in the molten char residues, which acted as physical barriers [51]. In the gas phase, on the one hand, the phosphorus-containing radical generated by the SAPC decomposition captured the active free radicals, inducing a quenching effect. On the other hand, SAPC inhibited the amount of flammable gases generated and promoted the release of non-flammable gases in the EP decomposition process, exerting asphyxiation and dilution effects [52]. In short, the significant improvement of flame retardancy and smoke suppression were mainly due to the catalytic expansion effect of SAPC and the good synergy between the acid source, carbonization agent, and blowing agent.

## 4. Conclusions

In summary, a macromolecular spirocyclic IFR SAPC with an integrated acid source, carbonization agent, and blowing agent was designed and synthesized. Its structure and performance were identified with FT-IR, ^1^H NMR and ^31^P NMR, and TGA. SAPC presented a good compatibility with EP, and the *T_g_* of the composites gradually increased with the increase of SAPC content. The EP composites with 8 wt % SAPC had an LOI of 30.3% and passed the UL-94 test with a V-0 rating. Interestingly, the EP/SAPC-8 presented a “blowout” phenomenon during the vertical combustion test. Moreover, the PHRR, THR and TSR values for EP/SAPC were obviously reduced. The TG-FTIR results showed that the amount of flammable volatile products from the EP composites’ decomposition of different degrees was suppressed after incorporating the SAPC, implying an attenuated flammability. Due to its multiple functions, SAPC may play a substantial role in improving fire safety and many other areas.

## Figures and Tables

**Table 1:** Infrared characteristic peak of the main pyrolysis product groups.

Wavenumber (cm^−1^)	Structure
3735,3650	–OH in H_2_O and phenols
3038,829	C–H in aromatic compounds
3540,3340	N–H in amine compounds
2970	–CH_3_, –CH_2_–in hydrocarbons
1744	C=O in aldehyde/ketone
1605,1510,1338	aromatic compounds
1257,1178	C–O in esters/ethers
